# From Ugly Duckling to Swan: Unexpected Identification from Cell-SELEX of an Anti-Annexin A2 Aptamer Targeting Tumors

**DOI:** 10.1371/journal.pone.0087002

**Published:** 2014-01-29

**Authors:** Agnes Cibiel, Nam Nguyen Quang, Karine Gombert, Benoit Thézé, Anikitos Garofalakis, Frédéric Ducongé

**Affiliations:** 1 Commissariat à l′Energie Atomique et aux Energies Alternatives (CEA), Direction des Sciences du Vivant (DSV), Intitut d’imagerie Biomédicale (I^2^ BM); 2 Institut National de la Santé et de la Recherche Médicale (INSERM) U1023, Laboratoire d’Imagerie Moléculaire Expérimentale, Orsay, France; 3 Université Paris Sud, Orsay, France; University of São Paulo, Brazil

## Abstract

**Background:**

Cell-SELEX is now widely used for the selection of aptamers against cell surface biomarkers. However, despite negative selection steps using mock cells, this method sometimes results in aptamers against undesirable targets that are expressed both on mock and targeted cells. Studying these junk aptamers might be useful for further applications than those originally envisaged.

**Methodology/Principal Findings:**

Cell-SELEX was performed to identify aptamers against CHO-K1 cells expressing human Endothelin type B receptor (ET_B_R). CHO-K1 cells were used for negative selection of aptamers. Several aptamers were identified but no one could discriminate between both cell lines. We decided to study one of these aptamers, named ACE4, and we identified that it binds to the Annexin A2, a protein overexpressed in many cancers. Radioactive binding assays and flow cytometry demonstrated that the aptamer was able to bind several cancer cell lines from different origins, particularly the MCF-7 cells. Fluorescence microscopy revealed it could be completely internalized in cells in 2 hours. Finally, the tumor targeting of the aptamer was evaluated *in vivo* in nude mice xenograft with MCF-7 cells using fluorescence diffuse optical tomography (fDOT) imaging. Three hours after intravenous injection, the aptamer demonstrated a significantly higher uptake in the tumor compared to a scramble sequence.

**Conclusions/Significance:**

Although aptamers could be selected during cell-SELEX against other targets than those initially intended, they represent a potential source of ligands for basic research, diagnoses and therapy. Here, studying such aptamers, we identify one with high affinity for Annexin A2 that could be a promising tool for biomedical application.

## Introduction

Nucleic acid aptamers are short oligonucleotides (<100 bases) that form three-dimensional structures capable of binding to a specific target with high affinity [1], [2]. Such structures are identified using a process of molecular evolution, known as SELEX for Systematic Evolution of Ligands by EXponential enrichment [2]. Basically, a combinatorial pool of sequences (from 10^12^ to 10^15^) is incubated with a target, and sequences bound to this target are recovered by a partitioning method before being amplified by PCR or RT-PCR and *in vitro* transcription (for DNA or RNA libraries, respectively). Then the pool is used for further rounds of partition/amplification and the enzymes used for the amplification can introduce some mutations leading to the apparition of new sequences that are capable of binding the target even more strongly than their parents. As a consequence, SELEX is often presented as *Darwinian* evolution in a test tube [3]. Only sequences with the best-inherited traits will survive and evolve, gradually leading to the accumulation in the population of the best nucleic acid structures to bind the target [4]–[6].

Since the invention of the SELEX process in 1990, aptamers have been selected against a wide variety of targets, from small compounds (amino acids, antibiotics…) to macromolecules (nucleic-acid structures, proteins…). They can rival with antibodies in terms of affinity, and like them, they can be used as inhibitors, activators or imaging probes [7]–[9]. As a consequence, they are extensively exploited as tools for research, diagnostic and also therapeutic applications. For instance, several aptamers are currently used to develop biosensors [10], [11], eight are currently enrolled in clinical trials, and one is already commercialized for the treatment of age-related macular degeneration [8], [12]. Furthermore, the straightforward modification and functionalization of aptamers make them ideal to address drugs, nanoparticles or contrast agents [13]–[20].

SELEX is mostly performed against a single purified target, but the method has recently been extended against heterogeneous complexes of targets and even whole-living cells [21]–[24]. The latter, usually named Cell-SELEX, is particularly useful to select aptamers against membrane proteins that are difficult to purify in their native conformation. Indeed, the three-dimensional structure of most membrane proteins is highly dependent on protein inclusion in lipid bilayers as well as their interaction with other membrane proteins or proteins from the extracellular matrix. However, thousands of proteins are present at the cell surface, which means that thousands of aptamers could theoretically co-evolve during Cell-SELEX. This could lead to decrease the speed of aptamer selection and to increase the difficulty in aptamer identification.

To circumvent this drawback, Cell-SELEX often performs negative selection steps using mock cells to favor the selection of aptamers against the targets that are specifically expressed on a cell of interest. Hence, we and other groups have used a specific cell line for negative selection steps (removing any aptamers that could bind to these cells) and the same cell line transformed to express a transmembrane protein for the positive selection of aptamers [25]–[29]. It favors the identification of aptamers against the expressed protein even when a high amount of other potential targets are present at the cell surface. Such strategy has also been used without prior knowledge of the targets to identify aptamers against biomarkers differentially expressed between different cell lines [30]–[33]. These aptamers could be further used to purify their targets before mass spectrometry analysis, in order to identify biomarkers of a specific cell phenotype [34]–[38].

However, we and other groups observed that the negative selection steps could be imperfect leading some undesirable aptamers to survive cell-SELEX although they bind to targets that are expressed both on mock and target cells [24], [25], [28]. These aptamers are usually put aside since they do not respond to the criteria defined at the time of the study. However, these aptamers have proven very successful adaptation properties since they are able to survive an evolutionary process designed to eliminate them. As a consequence, it might be interesting to study these deserving aptamers, which could be useful for further studies than those originally envisaged. Here, we investigated one of these undesirable aptamers and we demonstrated its potential value as tool for research or biomedical applications in cancer.

## Results

### Cell-SELEX Against CHO-K1 Cells Expressing Human Endothelin Type B Receptor (ET_B_R)

**Figure 1:**
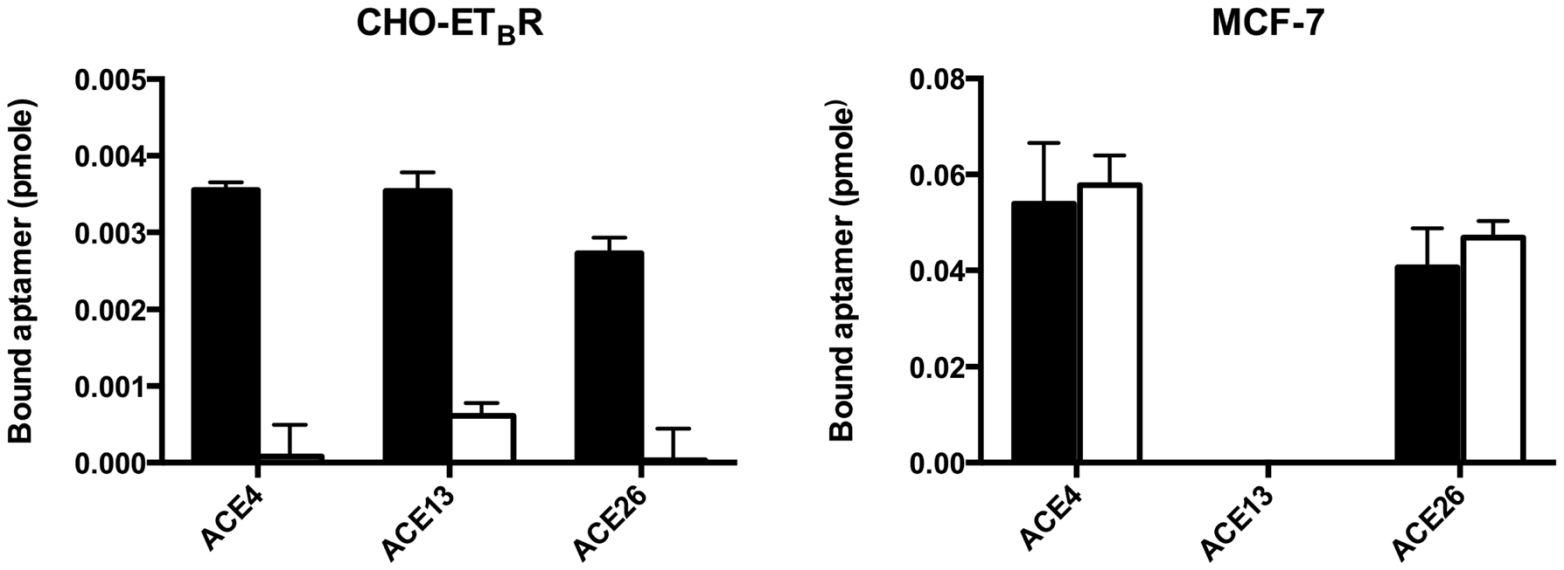
Binding of the ACE4, ACE13, and ACE26 aptamers on CHO-ET_B_R and MCF-7 cells. Binding assays were performed on CHO-ET_B_R and MCF-7 cells (left and right panel, respectively) using 25 nM of [^32^P] 5′-end radiolabeled aptamers. Black histograms represent the quantity of bound aptamers on untreated cells. White histograms represent the quantity of bound aptamers on cells pre-incubated with 100 nM of Endothelin-1 for 30 minutes before binding of aptamers. Error bars represent standard deviation of triplicate.

A cell-SELEX was performed against the hamster CHO-K1 cell line stably transformed to overexpress the endothelin receptor type B (CHO-ET_B_R). ET_B_R is a G protein-coupled receptor which activates a phosphatidylinositol-calcium second messenger system upon activation by Endothelins [39]. The wild type CHO-K1 cell line was used in negative-selection steps to discard any aptamer that could be selected against other membrane biomarkers than ET_B_R. Fifteen rounds of Cell-SELEX were performed as previously described [25]. 10, 000 sequences were analyzed for each round of Cell-SELEX using high-throughput sequencing (Table S1). A predominant amplification of some sequences was detected after 6 rounds where 10 sequences represented more than 1% of the pool. Some of these sequences disappeared during the additional cycles, while others were gradually amplified demonstrating that an evolutionary process took place. After 15 rounds of Cell-SELEX, 26 sequences of the pool represented each between 1% and 7% of the pool. The affinity of all these sequences was evaluated at 25 nM on CHO-K1 cells, CHO-ET_B_R cells and CHO-ET_B_R cells pre-incubated with Endothelin-1. The last condition induces a fast internalization of ET_B_R leading to its decrease from the cell surface [40]. Seven aptamers were identified and demonstrated a significantly higher binding to cells compared to a scramble sequence (Table S1). However, none of these aptamers seemed to bind ET_B_R because they also bound to wild type CHO-K1 cells. Nevertheless, three of these aptamers (ACE4, ACE13 and ACE26) were not able to bind to CHO-ET_B_R cells when the cells are pre-treated with Endothelin-1, suggesting these aptamers could interact with a target in close proximity or in interaction with ET_B_R (Fig. 1, left panel).

Competitive binding assays were performed to evaluate if these three aptamers could recognize a common target at the surface of CHO-ET_B_R cells. The binding of the ACE13 aptamer was not affected by the co-incubation with a mixture of the two other aptamers (Fig. S1). In contrast, the binding of the ACE4 and ACE26 aptamers was significantly reduced, suggesting that these two aptamers could bind to the same target although their predicted structures do not share any similarity (Fig. S2). The binding of these three aptamers was then evaluated on the human breast cancer cell line MCF-7 pre-incubated or not with Endothelin-1. MCF-7 cells are known to naturally express the type A and type B endothelin receptors [41] and represent a more physiological cellular model than transformed cells from hamster origin. No binding was observed with the ACE13 aptamer, suggesting it does not recognize a human form of its target or that its target is not present at the surface of MCF-7 cells (Fig. 1, right panel). In contrast, the ACE4 and ACE26 aptamers exhibited a binding ten times higher on MCF-7 cells compared to CHO cells. However, the pre-treatment with Endothelin-1 did not inhibit the binding of these aptamers, suggesting their target is not associated with ET_B_R at the surface of MCF-7 cells. Although our cell-SELEX strategy failed to identify aptamers against ET_B_R, we still decided to further study the ACE4 aptamer since its target seemed to be highly expressed at the surface of MCF-7 cells, which represent a well-known model of breast cancer. The ACE4 aptamer was chosen instead of ACE26 since both aptamers seemed to recognize the same target but ACE4 demonstrated a better affinity on cells than ACE26 (Fig. 1).

### Target Identification of ACE4 Aptamer

**Figure 2:**
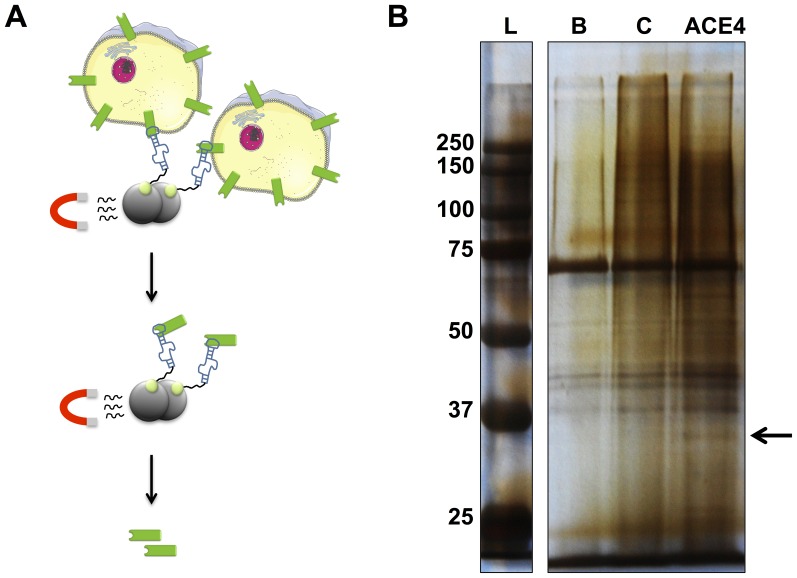
ACE4 aptamer-mediated target purification. A) Schematic representation of the method. Biotinylated aptamers (blue) bound to cells are attached to magnetic streptavidin beads and concentrated by magnetic field. Then, cells are lysed on beads and magnetic field is used to purify the target (green) bound to the aptamers before elution. B) After elution, the extracted proteins were separated by SDS-PAGE electrophoresis before being silver-stained in a polyacrylamide gel. Lane L, molecular marker. Lane B, extracted proteins with empty beads. Lane C, extracted proteins with a biotinylated scramble sequence. Lane ACE4, extracted proteins with the biotinylated ACE4 aptamer. The Arrow indicates a band with a substantial higher intensity in lane ACE4 compared to lane B and C. This band has been cut and analyzed by nano-LC-MS/MS Mass-spectrometry.

In order to elucidate what could be the target of the ACE4 aptamer, its affinity was evaluated on MCF-7 cells pre-treated with trypsin or proteinase K. Both treatments completely abolished the binding of the aptamer suggesting its target could be a membrane protein (data not shown). Different protocols have been used to isolate and identify the target protein of aptamers selected using cell-SELEX [34]–[36]. Here, we used a slightly modified protocol previously described by Berezovski *et al* [38] (Fig. 2A). The ACE4 aptamer was biotinylated at the 3′-end before incubation with MCF-7 cells. The cells strongly bound to the aptamer were collected at the surface of magnetic streptavidin beads before mild lysis in a solution containing 0.1% triton. After several washings, the proteins bound to the aptamers were eluted with urea, separated by SDS-PAGE electrophoresis, and visualized after silver staining. The same experiment was performed with the beads alone and with a biotinylated scramble sequence to distinguish proteins specifically bound to ACE4 from proteins non-specifically purified using this method. This purification was not really stringent and a high number of proteins were recovered. However, a band near 37 kD was clearly visualized following the aptamer purification while it was not observed after the purification with the control sequence or the beads alone (Fig. 2B). This band was excised before trypsin digestion and analysis by liquid chromatography-mass spectrometry (nano-LC-MS/MS). A protein match was obtained corresponding to Annexin A2.

**Figure 3:**
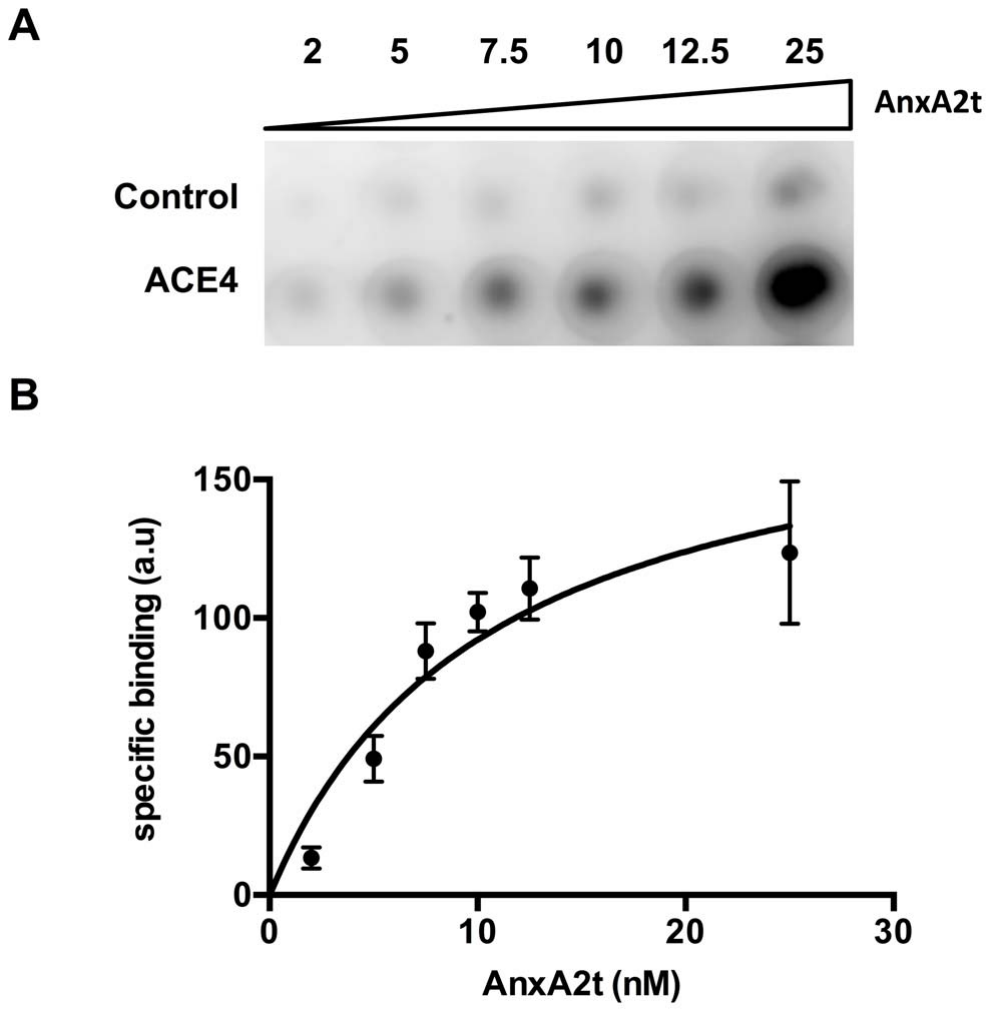
Filter binding assay of the ACE4 aptamer with purified hetero-tetramer Annexin A2 (AnxA2t). A) Different concentrations (from 2 to 25 nM) of the [^32^P] 5′-end radiolabeled ACE4 aptamer or scramble sequence were incubated with purified AnxA2t in the presence of a large excess of tRNA. Oligonucleotides bound to the protein were recovered on nitrocellulose filters and quantified by Phosphorimaging. B) Binding curve of the ACE4 aptamer on AnxA2t used for the evaluation of the binding constant (K_d_ = 10.5±4.6 nM). Error bars represent standard deviation of triplicate.

Annexin A2 exists both as a monomeric and as a hetero-tetrameric form. The monomer seems to be mostly intracellular and has been previously described as being a potential RNA binding protein [42], [43]. The hetero-tetramer (AnxA2t) has been localized to the plasma membrane and consists mostly of two subunits of Annexin A2 monomers with two subunits of p11 proteins (also known as S100A10) [44], [45]. Therefore, to verify that the ACE4 aptamer was selected against Annexin A2, its affinity was evaluated against a purified hetero-tetramer AnxA2t by filter binding assay (Fig. 3). The aptamer showed a tight binding with AnxA2t in comparison to a scramble sequence with an apparent K_d_ of 10.5±4.6 nM. Interestingly, the purified AnxA2t was from bovine origin suggesting that the aptamer can have a good cross species affinity.

### Binding of the ACE4 Aptamer on Different Cell Lines

**Figure 4:**
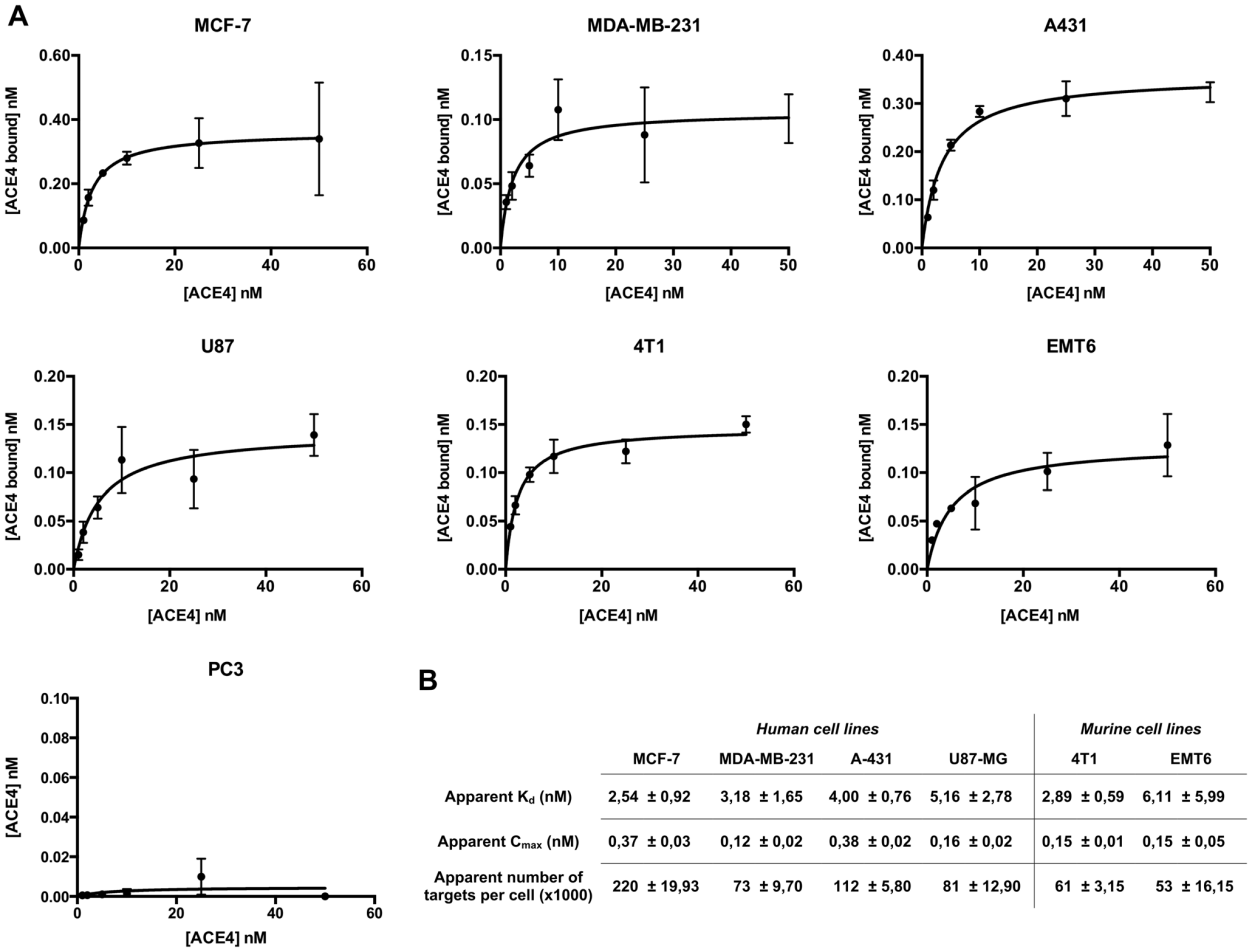
Binding of the ACE4 aptamer with different cancer cell lines. A) Binding curve of the ACE4 aptamer on different cancer cell lines. Different concentrations of [^32^P] 5′-end radiolabeled ACE4 aptamer or scramble sequences were incubated on cell monolayers. The graphics represent the quantity of aptamer bound to cells. The background non-specific binding values for a scramble sequence are subtracted for every data point. Error bars represent standard deviation of triplicate. B) The apparent K_d_, C_max_ and number of targets per cell for each cancer cell lines are calculated from A).

Several studies reported an increased expression of Annexin A2 in cancer tissues compared to normal tissues [45], [46]. Therefore, we evaluated the binding of [^32^P] 5′-end radiolabeled ACE4 aptamer on different cancer cell lines from human and mouse origins (Fig. 4A). The ACE4 aptamer do not bind PC3 cells established from human prostate carcinoma suggesting this cell line do not express Annexin A2. However, the ACE4 aptamer was able to bind all the other cancer cell lines tested with an apparent number of targets per cell ranged from 50,000 to 200,000 (Fig. 4B). All these cell lines were established from different cancer origins (MCF-7 and MDA-MB-231 cells from human breast cancer, A431 cells from human epidermoid carcinoma, U87 from human glioblastoma, 4T1 and EMT6 cells from murine mammary gland tumors). Interestingly, the aptamer exhibited a similar apparent K_d_ (2–6 nM) than the one measured with the purified AnxA2t (Fig. 3) confirming that AnxA2t should be the target of the aptamer. These results also confirm that the ACE4 aptamer has a good cross species affinity since it binds cells from human and mouse origins.

**Figure 5:**
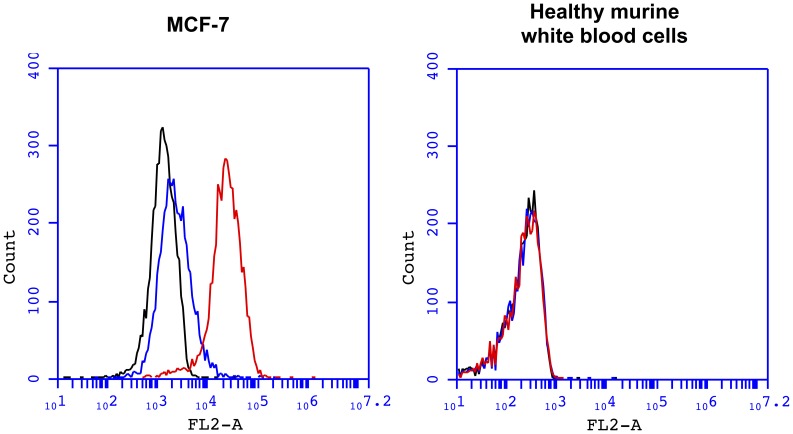
Comparison of the ACE4 aptamer binding on MCF-7 cancer cells and murine healthy white blood cells by flow cytometry. The binding of PE-labeled ACE4 aptamer or scramble sequence was measured by flow cytometry on MCF-7 cells and murine white blood cells (left and right panel, respectively). Black lines represent auto-fluorescence of cells, red lines represent the fluorescence of cells incubated with the PE-labeled ACE4 aptamer and blue lines represent the fluorescence of cells incubated with the PE-labeled scramble sequence. Counts represent the number of cells counted.

Using flow cytometry, the affinity of the ACE4 aptamer was further compared between MCF-7 cells and healthy white blood cells extracted from the blood of a nude mouse. The fluorescence intensity of MCF-7 cells showed a significant increase when they are incubated with the fluorescently labeled ACE4 aptamer compared to a scramble sequence (Fig. 5 left panel). On the contrary, no binding was observed with white blood cells (Fig. 5 right panel). These results demonstrate that the MCF-7 cancer cells express a significantly higher level of Annexin A2 at their surface compared to healthy white blood cells.

### ACE4 Aptamer Studies by Fluorescent Microscopy

**Figure 6:**
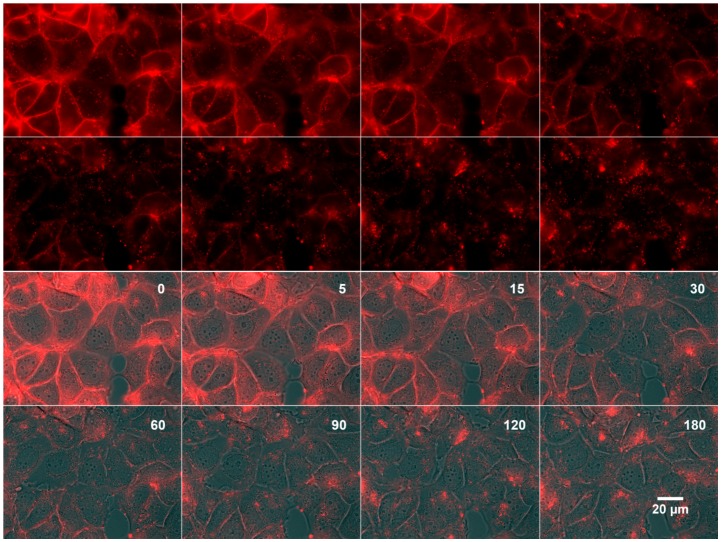
Time-lapse imaging over 3 hours of the ACE4 aptamer internalization in MCF7 cells. The fluorescently labeled ACE4 aptamer was incubated with MCF-7 cells before washing and fluorescent time-lapse imaging. The first two rows display the red fluorescent channel only. The two second ones are a merge of the corresponding fluorescent and brightfield channels. The elapsed time is expressed in minutes and recorded on each merged image only.

Several studies demonstrated that aptamers could be internalized inside cells following their binding to cell surface proteins [7], [17], [47], [48]. In order to evaluate if the ACE4 aptamer could have this behavior, its subcellular localization was studied over time using fluorescence microscopy. The fluorescently labeled aptamer was first incubated 30 minutes with MCF-7 cells. Then, unbound aptamers were removed with several washings before time-lapse imaging (Fig. 6 and Video S1). Most of the ACE4 aptamer was localized at the cell surface after 30 minutes of incubation and only low signal was detected within intra-cellular vesicles. Then, the signal disappeared at the cell surface with complete loss after 2 hours while it was increasing inside intracellular vesicles. Finally, fluorescence remained trapped in cells at least for 22 hours (Video S1). In the same conditions, no fluorescence signal above the background was detected with the scramble sequence (data not shown). These results provide strong evidence that the ACE4 aptamer could be efficiently internalized inside cells.

### *In vivo* Targeting of ACE4 Aptamer to MCF-7 Tumor Xenograft

Tumor targeting of the ACE4 aptamer was then studied in nude mice bearing subcutaneous tumor xenografts of MCF-7 cells. The fluorescently labeled ACE4 aptamer was injected intravenously in the caudal vein and its biodistribution was monitored using planar near infrared (NIR) fluorescence imaging during 3 hours. Just after injection, the fluorescence signal was diffused in the whole body with a highest contrast in blood vessels which disappeared rapidly in few minutes suggesting a fast distribution from blood to tissue (Fig. S3). A rapid elimination by the urinary pathway was observed with an increasing signal in the kidneys over time and a very high signal inside the bladder after three hours. A low signal in the liver was also noticed three hours post-injection, suggesting a partial elimination by the hepato-biliary pathway (Fig. S3B). The same pattern of biodistribution was observed for a scramble sequence, but a highest contrast between the tumor and the surrounding tissue was observed with the ACE4 aptamer (Fig. S3A).

**Figure 7:**
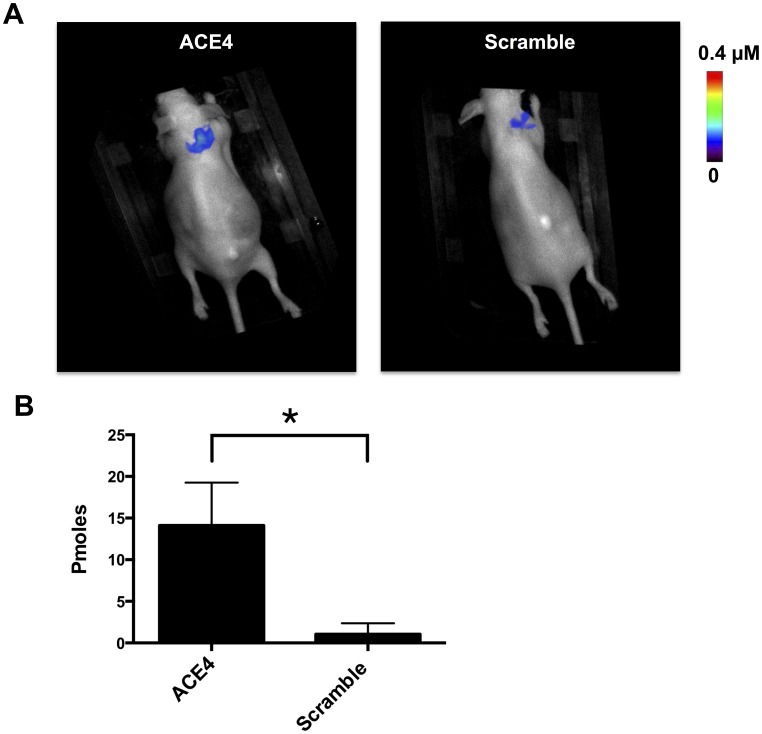
Tumor targeting of the ACE4 aptamer and scramble sequence measured by *in vivo* fluorescence diffuse optical tomography (fDOT) imaging. 3-intravenous injection, a fDOT imaging scan was performed in the tumor zone. A) Visualization in the Bird’s-eye view of the 3D reconstructed fluorescent signal in the tumor (color LUT) overlaid on the white light image of the mouse for the ACE4 aptamer (left panel) and the control sequence (right panel). B) Quantity of oligonucleotide inside tumors calculated from the 3D fluorescent signal A). Error bars represent standard deviation of triplicate. *P<0.05.

The fluorescence signal in the tumor was then quantified by fluorescence diffuse optical tomography (fDOT) imaging (Fig. 7). This technique, also known as fluorescence molecular tomography (FMT), operates in a transillumination excitation mode, and uses sophisticated reconstruction algorithms to reconstruct in 3D and quantify fluorescence signal inside a small animal [49], [50]. Three hours after injection, the quantity of ACE4 aptamer in the tumor was around fourteen times higher compared to the scramble sequence (14.11±2.97 pmoles (≈1% of injected dose) compared to 1.05±0.75%, respectively). To verify that the imaging results were not affected by degradation of the sequences by nucleases, their stabilities were compared in 10% serum at 37°C (Fig. S4). Both the ACE4 aptamer and the scramble sequence displayed a similar resistance to nucleases with around 40% of degradation after three hours. These results suggest that the difference in tumor imaging between the ACE4 aptamer and the scramble sequence is not due to a better resistance of the aptamer against nucleases. Altogether, these results suggest that the ACE4 aptamer represents a promising tool for the *in vivo* imaging of tumors overexpressing the Annexin A2.

## Discussion

Cells express a high diversity of targets at their surface. As a consequence they represent a more complex selection support than a chromatography matrix or a nitrocellulose filter that are usually used for classical SELEX. Therefore, despite the negative selection steps, aptamers are sometimes selected against cell surface biomarkers that are expressed both on mock and target cells [24], [25], [28]. This phenomenon could be explained by some bias of Cell-SELEX which are difficult to unbalance. For instance, it is assumed that, despite negative selection, Cell-SELEX may favor the emergence of the aptamers that bind to highly abundant targets or recognize proteins with favorable conformations to generate high affinity aptamers. In our case, it seems that Annexin A2 was not particularly abundant at the surface of CHO cells during SELEX (≈30,000 apparent targets per cell). On the contrary, it has been demonstrated that Annexin A2 has a role inside cells as a RNA binding protein and thus it might be favorable to generate aptamers.

Whatever the reason why undesirable aptamers are selected against other targets than those expected during a cell SELEX, they represent an invaluable source of ligands. Of course using such aptamers needs first to identify their target before foreseeing the applications or research areas in which they could be used. However, several studies have already demonstrated that aptamers could be used to purify their targets by chromatography before identification by mass spectrometry [34]–[38]. Although these methods have still to be improved, we successfully identified Annexin A2 as being the target of the ACE4 aptamer using such approaches.

Annexin A2 is up-regulated in various tumor types and plays multiple roles in regulating cellular functions such as angiogenesis, proliferation, cell migration and adhesion [45]. Accordingly, it would be interesting to determine if the ACE4 aptamer could have a neutralizing effect on the protein. Furthermore, several aptamers selected against cell surface proteins have already been used to address drugs or contrast agents, including nanoparticles or siRNA [7], [17], [47], [48]. The ACE4 aptamer might be useful for such purposes since it could be internalized inside cells upon binding to Annexin A2 and has already demonstrated promising *in vivo* tumor targeting by *in vivo* imaging.

In conclusion, our data reveal that undesirable aptamers isolated during cell-SELEX deserve to be studied although they are selected against other targets than those initially intended. These recycled aptamers could be particularly useful for basic research, diagnosis and therapy.

## Materials and Methods

### Ethics Statement

All animal use procedures were in strict accordance with the recommendations of the European Community (86/609/CEE) and the French National Committee (décret 87/848) for the care and use of laboratory animals. Ethics committee of CETEA – CEA DSV (Comité d’Ethique en Expérimentation Animale (CETEA), de la Direction des Sciences du Vivant (DSV) du Commissariat à l’Energie Atomique et aux énergies alternatives (CEA)) approved the study (ref: 12-093).

### Reagents

Except when specified, reagents used for molecular and cellular biology are from Life Technologies, chemical reagents from Sigma-Aldrich and chemically synthesized oligonucleotides from Eurogentec.

### Cells

CHO-K1 (ECACC, Wiltshire, UK) and CHO-ETBR cells (CHO-K1 cells stably transfected with vector expressing human ET_B_R [40]) were donated by the laboratory of Frédéric Ducancel (CEA, Saclay, France). U87-MG cells (ATCC, Manassas, VA) were donated by the laboratory of Andreas Jacobs (Max Plank Institute, Köln, Germany). 4T1 and EMT6 cells (ATCC, Manassas, VA) were donated by the laboratory of Lina Bolotine (Research Centre for Automatic Control (CRAN), Nancy-University, UMR CNRS, France). MDA-MB-231, A-431, MCF-7 and PC3 cells were purchased from ATCC (Manassas, VA).

CHO cells were grown in Nutrient Mixture F-12 HAM supplemented with 10% Foetal Bovin Serum (FBS). MCF-7 cells were grown in RPMI media supplemented with 10% FBS. A431, MDA-MB-231, U87-MG, 4T1, and EMT6 cells were grown in DMEM media with 10% FBS. All the cell types were grown at 37°C in a 5% CO_2_ atmosphere.

### Animal Models

All used mice were female nude mice weighing approximately 23 g and housed under standard conditions with food and water *ad libitum*. One week before the implantation of cancer cells, pellet of 17-β estradiol (0.72 mg, Innovative research of america) were subcutaneously transplanted in mice using precision trochar (Innovative research of america) according to provider instructions. Then, mice were subcutaneously injected between shoulder blades with 10^7^ MCF-7 cells in a volume of 200 µl of Matrigel (BD Bioscience, Le Pont de Claix, France) and phosphate- buffered saline (PBS) (50∶50). Tumors were then allowed to grow for several weeks until a size around 300 mm^3^ before *in vivo* imaging experiments. During each injection and imaging experiments, mice were anesthetized with isoflurane–1.25% in a 1∶3 mixture of O_2_ and air.

### Cell-SELEX Against Cells Over-expressing ETBR

Cell-SELEX was performed against the CHO-K1 cell line stably transformed to over-express endothelin receptor type B (CHO-ET_B_R) using a protocol that we previously described [25]. The 2′F-Py RNA library used for selection consisted of a 50-nucleotide random region (N50) flanked by two constant regions: 5′-GGGAGAUGAUCCGUUGAUGCGAG-(N50)-AAGUCGUCGUUCGUAGGCAGAAUC-3′. This library was generated by transcribing around 10^14^ DNA double strand templates in the presence of 1 mM ATP, GTP, 2′F-Py-UTP and 2′F-Py-CTP (Trilink Biotechnologies) and a mutant form of T7 RNA polymerase (T7Y639F, kind gift of R. Souza) [51] followed by DNase treatment and PAGE purification. 2′F-Py RNAs (2 nmol) were heated at 85°C for 5 min in 3 ml of RPMI 1640, snap-cooled on ice for 2 min and allowed to warm up to 37°C before two negative selection steps against CHO-K1 cells (incubation during 30 minutes with 10^7^ adherent CHO-K1 cells, unbound sequences were recovered and incubated again during 30 minutes with 10^7^ adherent CHO-K1 cells). Then unbound sequences are recovered and used for the selection step by incubation with 10^7^ adherent CHO-ET_B_R cells during 30 minutes. After several washings with RPMI 1640, bound sequences were recovered by Trizol extraction (Sigma-Aldrich) before being amplified by RT-PCR (using the primers P60 (5′-GATTCTGCCTACGAACGACGACTT-3′) and P70 (5′-CTCGAGTAATACGACTCACTATAGGGAGATGATCCGTTGATGCGAG-3′) and *in vitro* transcription. After DNase treatment, 2′F-Py RNAs were PAGE purified before another round of negative-selection and selection. During the selection process, we progressively increased the selection pressure by increasing the number of washings (from two up to five), adding nonspecific yeast tRNA competitor (100 µg/ml in the last four cycles), decreasing the incubation time (from 30 to 10 min) and decreasing the number of cells exposed to the aptamers (from 10^7^ to 5×10^6^).

After 15 rounds of cell-SELEX, the pool for each round was sequenced with the Genome Analyzer II of Illumina according to provider’s instructions. Basically, a PCR aliquot of each round was amplified by PCR using primers containing specific adapter sequences and indexing sequences to allow the multiplexing of all samples in a single sequencing run. Each sample were gel purified, mixed at equimolar concentration and then sequenced onto a single lane of the microfluidic cell using 80 sequencing cycles in single read. Millions sequences per round of cell-SELEX were obtained. A quality score filter was used and sequences were sorted thanks to indexing sequences. Finally, around 1 million sequences per round of cell-SELEX were obtained in fastq files. Deep sequencing data were loaded on Galaxy project platform (http://galaxyproject.org/) and are available at https://usegalaxy.org/u/nicolas1988/h/rawreadseqetbr under the accession number RawReadSeqETBR. 10,000 sequences were selected randomly per round of cell-SELEX and then aligned. Group of close sequences (few mutations between them) were identified, named and counted.

### 2′F-Py RNA Aptamer Synthesis

Plasmids containing the aptamer sequences or chemically synthesized DNA aptamer sequence were used as template for PCR using primers P60 and P70 before being *in vitro* transcribed and purified according to the previously described protocol for the cell-SELEX. For cytometry, the primer G-P60 (′5-TAGGCAGTCACGCTGGGGCAATGCGATTCTGCCTACGAACGACGACTT-3′) was used instead of P60 to produce 2′F-Py RNA aptamers with a 24 nt extension at the 3′ end.

The sequences of the identified aptamers are provided below as well as the scramble sequence that we randomly designed to be used as negative control:

ACE1′5-GGGAGAUGAUCCGUUGAUGCGAGGCGACAAGGAGTATTTTTGCCTTGTCCGGCCCTCACCTCGGGTCACGTGGAAGUCGUCGUUCGUAGGCAGAAUC-3′;

ACE45′-GGGAGAUGAUCCGUUGAUGCGAGGGAACGCAAGAACUGAGGCCAUGAGGCGCCUUCCCUUGCUCAGGACGCAAGUCGUCGUUCGUAGGCAGAAUC-3′;

ACE11′5-GGGAGAUGAUCCGUUGAUGCGAGCAATGCGTTACAACAACGGACGTGCCTGCCAGCGCTATACCCAAGUCGUCGUUCGUAGGCAGAAUC-3′;

ACE13′5-GGGAGAUGAUCCGUUGAUGCGAGCAGAGCCGCTGGCGACTTATTCCAACAGTCGCCCCCACAACCCTTGTCCCAAGUCGUCGUUCGUAGGCAGAAUC-3′;

ACE16′5-GGGAGAUGAUCCGUUGAUGCGAGACCCAAGTTGGGACCCATCCACGCCTCAGGCCGTTTGGCGCCGACCCAGTAAGUCGUCGUUCGUAGGCAGAAUC-3′;

ACE23′5-GGGAGAUGAUCCGUUGAUGCGAGCTACATGCCGCTAGTCTGCAGACCCTCGCAATCAAGTCTACCAGCACGCAAGUCGUCGUUCGUAGGCAGAAUC-3′;

ACE26′5-GGGAGAUGAUCCGUUGAUGCGAGAGCTAGGCCGCAAGGTGCCTCAACGCCATCTGAGTGCCGACCCGATCGCAAGUCGUCGUUCGUAGGCAGAAUC-3′;

Scramble sequence′5-GGGAGAUGAUCCGUUGAUGCGAGGAUCCCUACGACCUCGUAGCACACACAUAGGUGCACUCACCCGGCUGACCAAGUCGUCGUUCGUAGGCAGAAUC-3′.

### Aptamer-based Target Purification and Identification

The ACE4 aptamer and the scramble sequence were 3′ end biotinylated using biotinamidocaproyl hydrazide (Pierce) as previously described [52]. MCF-7 cells were washed twice with PBS and harvested with PBS containing 5 mM EDTA. A total of 5.10^6^ cells were incubated for 30 min on ice in 1 mL of D-PBS containing 1 g/L D-glucose, 36 mg/L sodium pyruvate, 1 mM calcium, 0.5 mM magnesium Dulbecco’s Phosphate Buffered Saline (D-PBS) and supplemented with 20 nM biotinylated ACE4 or scramble sequence in the presence of tRNA, ssDNA, and polyinosine (100 µg/mL each). Cells were washed twice by centrifugation (5 min, 300 g) with 1 mL of D-PBS supplemented with BSA (0.5 µg/µL). Then they were incubated in 1 mL of the same buffer with 1 mg of Dynabeads M-280 streptavidin (Invitrogen) for 15 min on ice. Bead-cell-aptamer complexes were recovered using a magnetic stand and washed twice with D-PBS. The beads were suspended in 1 mL of lysis buffer (D-PBS containing 0.1% triton X-100 and tRNA, ssDNA, and polyinosine at 100 µg/mL each). After 30 min on ice, the cell lysate was recovered and the beads washed twice with lysis buffer without nucleic acid competitors. Finally, protein targets were eluted from the aptamer-bead complexes by adding 30 µL of 8 M urea and incubation for 15 min at 60°C. The supernatant was then recovered and loaded on 10% SDS-PAGE gels before silver staining (Proteosilver stain kit, Sigma). ACE4-specific bands were cut and analyzed by nano-LC-MS/MS.

### Binding of Radioactive Aptamers on Adherent Cells

First, 2′F-Py RNAs were dephosphorylated at the 5′-end using antartic phosphatase (New England Biolabs) before 5′-[^32^P] labeling (3×10^3^ Bq.pmole^−1^) using T4 kinase. All binding experiments were performed in triplicate on 24-well plates using a Microlab Starlet automate (Hamilton). Radioactive aptamers at different concentrations were incubated for 15 min at 37°C on 80% confluent cell monolayer in 200 µL of RPMI 1640 containing 100 µg/ml of tRNA as a nonspecific competitor. After five washings with 500 µL of culture medium, bound sequences were recovered in 200 µL of lysis buffer (50 mM Hepes PH 7.5, 150 mM NaCl, 10% glycerol, 1% Triton X100, 2 mM MgCl2 and 5 mM EGTA) and the radioactivity counted. For all binding experiments, specific binding was measured by subtracting background values obtained with a scramble sequence in the same conditions.

Apparent *K_d_* values and the total target concentration (*C_max_*) were determined by fitting binding curve with Graph Prism 6 using One site specific binding model. The apparent number of targets per cell was calculated multiplying the Avogadro number per (C_max_ × volume of incubation)/number of cells per well.

### Binding of Radioactive Aptamers with Purified AnxA2t

1pmole of 5′- [^32^P] labeled ACE4 aptamer and scramble sequence (3×10^3^ Bq.pmole^−1^) were incubated with different concentrations (0, 2, 5, 7.5, 10, 12.5, 25 nM) of a hetero-tetramer form of Annexin A2 (AnxA2t) purified from bovine lung (Interchim, France, ref: A80109B). The incubation was performed in 200 µl of RPMI 1640 in the presence of 3 µg of yeast tRNA during 30 min at room temperature. Then the mixture was passed through a nitrocellulose filter (HAWP 0,45 μ, Millipore) to recover oligonucleotides bound to AnxA2t. After extensive washing with RPMI 1640, the filter was dried before analysis and quantification by Phosphorimaging using a STORM apparatus (GE healthcare). Specific binding curve was obtained by subtracting background values obtained with a scramble sequence for every data point. Apparent *K_d_* values and the total target concentration (*C_max_*) were determined by fitting binding curve with GraphPad Prism 6 using One site specific binding model.

### Flow Cytometry

For cytometry experiments, RPMI 1640 without red phenol was used. Elongated ACE4 aptamer and scramble sequence were hybridized to a 5′ end biotinylated DNA oligonucleotide (′5-CTC-GAG-TAG-GCA-GTC-ACG-CTG-GGG-CAA-TGC-3′) before labeling with Phycoerythrin-conjugated streptavidin (SA-PE, Interchim) without further purification as previously described by Li and al [53]. 10 nM of PE labeled ACE4 and scramble sequence were incubated during 30 minutes at 0°C with 100,000 cells in 200 µl of RPMI 1640 containing 100 µg/ml of yeast tRNA. Unbound sequences were removed by two washings with 5 mL of RPMI 1640 media using 5 min centrifugation at 300 g. Then cells were suspended into 300 µL of RPMI 1640 and kept on ice before being analyzed on the FL-2 channel with BD Accuri C6 flow cytometer (BD biosciences). To obtain murine white blood cells, 100 µL of fresh blood sample was immediately mixed on ice with 20 µL of EDTA 20 mM, pH 7. Then, 1 mL of NH_4_Cl was added to lyse red blood cells during 10 min on ice. Finally, white blood cells were washed with PBS, centrifuged (5 min, 300 g) to remove remaining red blood cells and suspended in RPMI 1640 before incubation with oligonucleotides. For MCF-7 cells, adherent cell monolayer was detached with Versene. Versene was removed after centrifugation (5 min, 300 g) and cells were suspended in RPMI 1640 before incubation with oligonucleotides.

### Fluorescent Microscopy

The ACE4 aptamer and the scramble sequence were labeled using the Ulysis™ AlexaFluor 546 Nucleic Acid Labeling Kit (Life Technologies) according to the provider instructions. The relative efficiency of the labeling reaction was evaluated by spectrophotometry and calculated as the ratio between the concentration of dye and the concentration of aptamer. The protocol was optimized to obtain a ratio of approximately one dye per aptamer. Unconjugated dyes were removed using Bio-Spin columns containing Bio-Gel® P-6 (Biorad) pre-equilibrated with saline-sodium citrate (SSC) buffer containing 3 mM Mg^2+^. For time-lapse imaging, 125,000 MCF-7 cells were seeded on collagen coated 35 mm glass bottom petri dishes (Mattek). After 72 h of growth in RPMI with 10% FBS and without phenol red, medium was renewed and supplemented with tRNA at a final concentration of 100 µg/mL for nonspecific binding saturation. Fluorescently labeled ACE4 or scramble sequences were added at a final concentration of 10 nM and incubated on cells at 37°C for 30 minutes. After extensive washings with D-PBS, image acquisition was performed during 22 h under an epifluorescent AxioObserver Z1 microscope (Zeiss) equipped with an integrated incubation chamber.

### *In vivo* Imaging Experiments

The ACE4 aptamer and the scramble sequence were labeled using the Ulysis™ AlexaFluor 680 Nucleic Acid Labeling Kit (Life Technologies) as previously described. The fluorescent ACE4 aptamer or scramble sequence were intravenously injected at a dose of 50 pmoles/g in anesthetized mice bearing subcutaneous tumor xenografts from MCF-7 cells (n = 3). Planar near infra-red (NIR) fluorescence images (dorsal and frontal) were acquired before and after the injection using the planar imaging option of the TomoFluo3D fluorescent tomographic system (developed by CEA/LETI and Cyberstar) [49]. Measurements were performed at different time points using a 700 nm long pass filter (Schott-RG9, Itos, Germany) and by setting the exposure time at 100 ms.

Three hours post-injection, a transillumination 685nm-laser scan (5×5 points) was performed at the tumor level. The camera images of the different source positions were used for the reconstruction of the fluorescent signal according to a procedure that is described in detail elsewhere [49]. The output of the optical reconstruction was given in 3D matrices of fluorescent signal with a voxel resolution of 0.67×0.67×1 mm^3^ (X, Y, Z). Visualization and quantification of the fDOT signal was performed using the Brainvisa medical imaging processing software (http://brainvisa.info/index_f.html) using a previously reported calibration curve [50]. To determine statistical significance of tumor uptake between ACE4 aptamer and scramble sequence, mean fluorescence values were subjected to unpaired t-test analysis using GraphPad Prism 6 with a confidence interval of 95% (n = 3).

### Nuclease Resistance Assay

The ACE4 aptamer and the scramble sequence were labeled using the Ulysis™ AlexaFluor 680 Nucleic Acid Labeling Kit (Life Technologies). The fluorescent ACE4 aptamer or scramble sequence were incubated at 37°C for different time in RPMI 1640 containing 10% calf serum. Then, 2′F-Py RNA was extracted by Trizol before being loaded on 3% agarose gel. The fluorescent signal in the gel was revealed by the TomoFluo3D fluorescent tomographic system. The percentage of full-length oligonucleotides was measured by comparing the intensity of the bands corresponding to the migration of intact 2′F-Py RNA normalized by the band of full-length 2′F-Py RNA without incubation with serum.

## Supporting Information

Figure S1 **Competitive binding between the ACE4, ACE13, and ACE26 aptamers on CHO-ET_B_R cells.** Binding assays were performed on CHO-ET_B_R cells using 10 nM of [^32^P] 5′-end radiolabeled aptamers. Histograms represent the quantity of bound aptamers on cells in the absence (black) or in the presence of 100 nM (white) of the two other aptamers as unlabeled specific competitor. Error bars represent standard deviation of triplicate.
(TIFF)

Figure S2 **Comparison of secondary structures prediction for the ACE4 and the ACE26 aptamers.** A) Secondary structure prediction for the ACE4 and ACE26 aptamers using Mfold web server (http://mfold.rna.albany.edu/?q=mfold/RNA-Folding-Form) for nucleic acid folding and hybridization prediction (*Nucleic Acids Res.* (2003) **31 (13)**, 3406-15,). B) Predicted alignment of ACE4 and ACE26 aptamers based on their predicted structures using CARNA - alignment of RNA structure ensembles program (http://rna.informatik.uni-freiburg.de) (Nucleic Acids Research, 40 no. W1 pp. W49–W53, 2012).
(TIFF)

Figure S3 **Biodistribution of the ACE4 aptamer and scramble sequence measured by** ***in vivo*** **planar near infrared (NIR) fluorescence imaging into nude mice bearing subcutaneous tumor xenografts from MCF-7 cells.** Planar NIR fluorescence images were acquired at different times after the intravenous injection of fluorescently labeled ACE4 aptamer or control sequence into nude mice bearing tumor xenografts from MCF-7 cells. A) Dorsal view of the fluorescent ACE4 aptamer and scramble sequence at different times (min^−1^) post-intravenous injection. Arrows indicate T: tumor and K: kidney. B) Ventral view of the fluorescent ACE4 aptamer 15 min and 180 min post-injection. Arrows indicate L: liver and B: bladder.
(TIFF)

Figure S4 **Nuclease resistance of the ACE4 aptamer and the scramble sequence in 10% serum.** A) The fluorescent ACE4 aptamer (upper panel) and scramble sequence (lower panel) were incubated at 37°C for different times in 10% serum before being analyzed by electrophoresis on a 3% agarose gel. B) Evolution of the percentage of full-length oligonucleotides over time measured from A).
(TIFF)

Table S1 **Evolution of sequences during the rounds of cell-SELEX.** 135 sequences have been found at more than 0,05% of the pool during at least one round of cell-SELEX. They are ranked from their relative abundance in the latest round and their numbers per 10,000 sequences of the pool are presented for each round. Binding at 25 nM on CHO-K1 cells, CHO-ET_B_R cells and CHO-ET_B_R cells pre-incubated with Endothelin-1 was evaluated for the sequences that represent more than 1% of the pool after the last round,+indicates binding, – indicates no significant binding compared to a scramble sequence.
(TIFF)

Video S1 **Movie of the ACE4 aptamer internalization in MCF7 cells over 22 hours.** The Fluorescently labeled ACE4 aptamer was incubated with MCF-7 cells before washing and fluorescent time-lapse imaging. The images are a merge of the related red fluorescent and brightfield channels. The elapsed time (in minutes) and scale bar are displayed on the top and bottom right sides of the images, respectively.
(MP4)
