# Supplementary figures and images for: From Ugly Duckling to Swan: Unexpected Identification from Cell-SELEX of an Anti-Annexin A2 Aptamer Targeting Tumors
Source: PLoS One. 2014 Jan 29;9(1):e87002. doi: 10.1371/journal.pone.0087002 (PMC3906106; doi:10.1371/journal.pone.0087002)

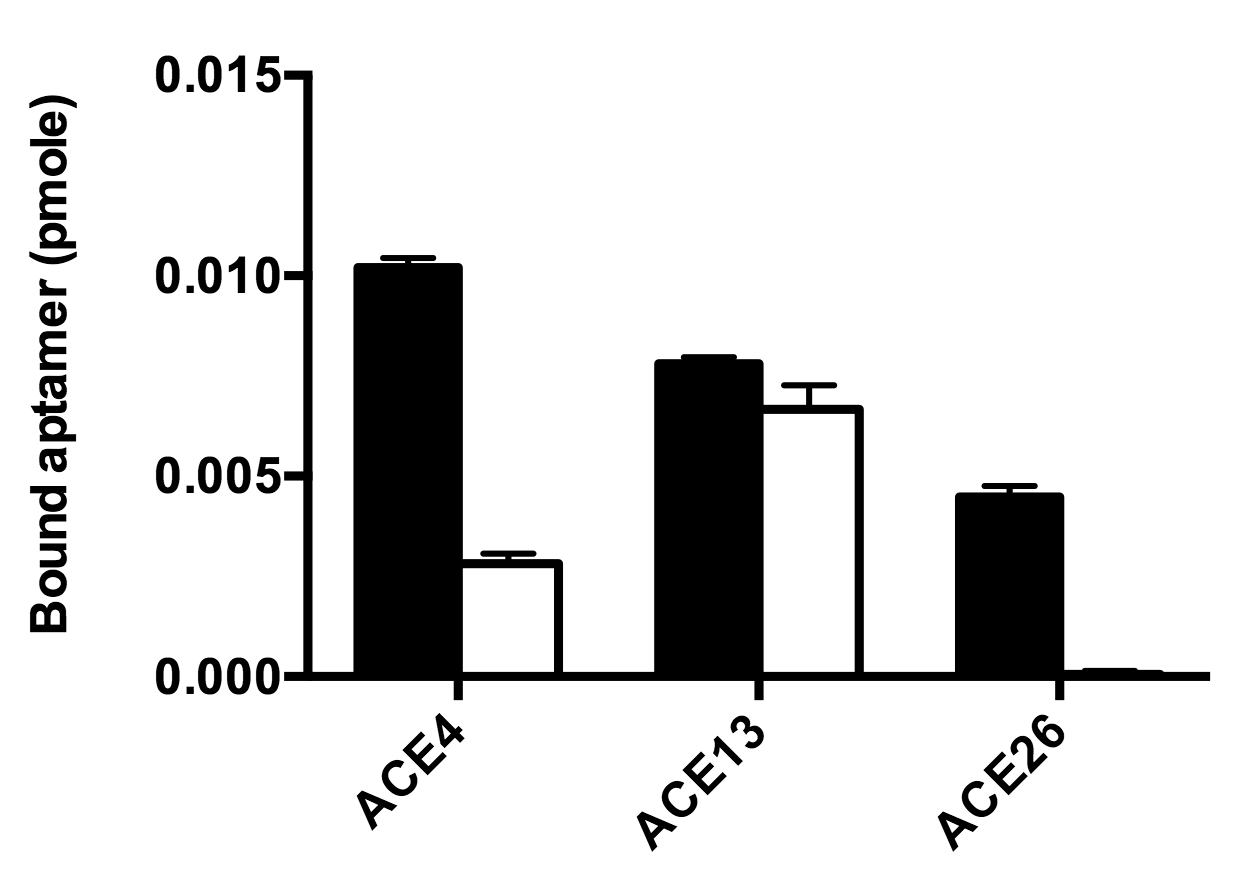

Supplement: Figure S1 — Competitive binding between the ACE4, ACE13, and ACE26 aptamers on CHO-ETBR cells. Binding assays were performed on CHO-ETBR cells using 10 nM of [32P] 5′-end radiolabeled aptamers. Histograms represent the quantity of bound aptamers on cells in the absence (black) or in the presence of 100 nM (white) of the two other aptamers as unlabeled specific competitor. Error bars represent standard deviation of triplicate. (TIFF) [file pone.0087002.s001.tiff]

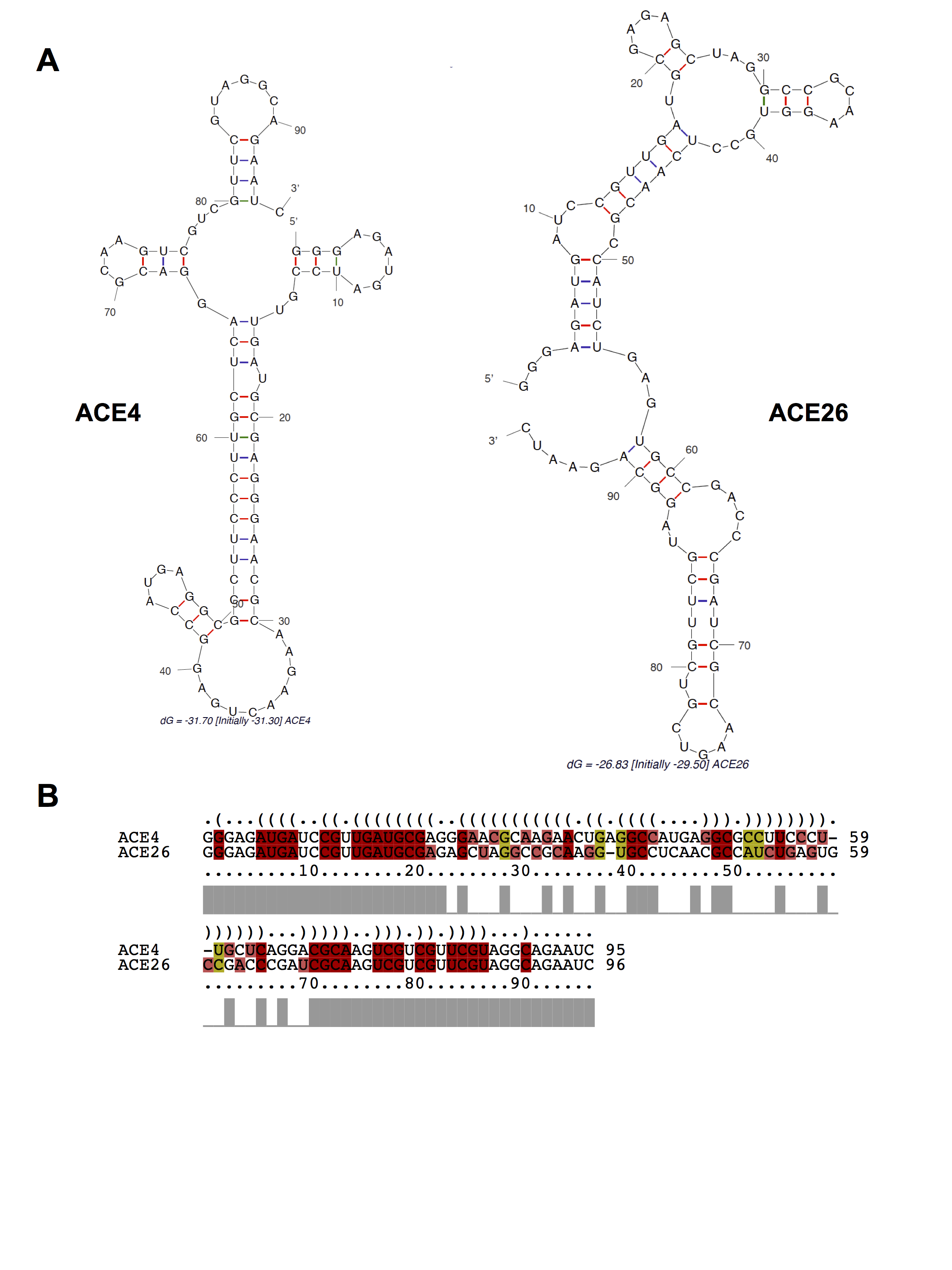

Supplement: Figure S2 — Comparison of secondary structures prediction for the ACE4 and the ACE26 aptamers. A) Secondary structure prediction for the ACE4 and ACE26 aptamers using Mfold web server (http://mfold.rna.albany.edu/?q=mfold/RNA-Folding-Form) for nucleic acid folding and hybridization prediction (Nucleic Acids Res. (2003) 31 (13), 3406-15,). B) Predicted alignment of ACE4 and ACE26 aptamers based on their predicted structures using CARNA - alignment of RNA structure ensembles program (http://rna.informatik.uni-freiburg.de) (Nucleic Acids Research, 40 no. W1 pp. W49–W53, 2012). (TIFF) [file pone.0087002.s002.tiff]

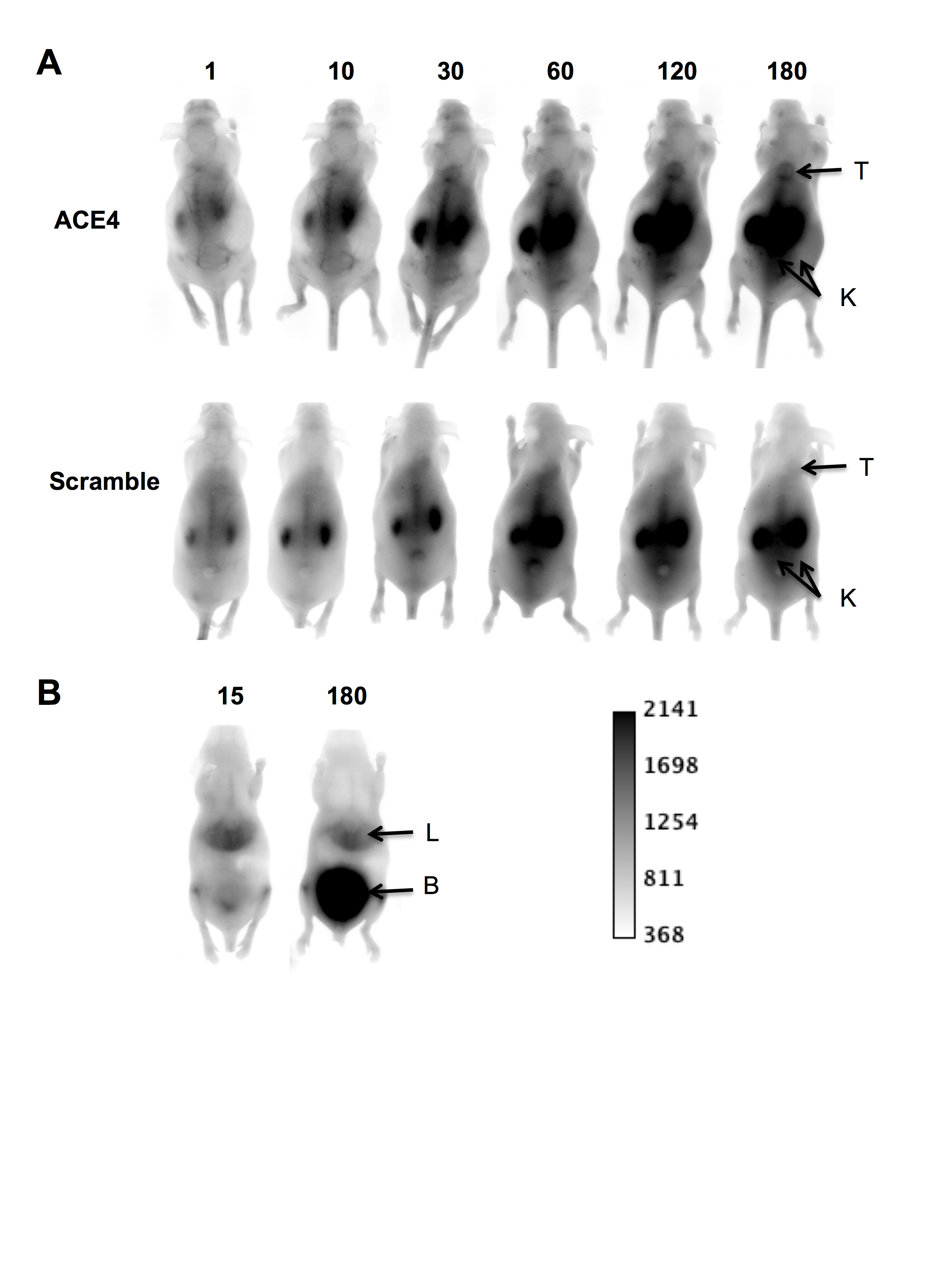

Supplement: Figure S3 — Biodistribution of the ACE4 aptamer and scramble sequence measured by in vivo planar near infrared (NIR) fluorescence imaging into nude mice bearing subcutaneous tumor xenografts from MCF-7 cells. Planar NIR fluorescence images were acquired at different times after the intravenous injection of fluorescently labeled ACE4 aptamer or control sequence into nude mice bearing tumor xenografts from MCF-7 cells. A) Dorsal view of the fluorescent ACE4 aptamer and scramble sequence at different times (min−1) post-intravenous injection. Arrows indicate T: tumor and K: kidney. B) Ventral view of the fluorescent ACE4 aptamer 15 min and 180 min post-injection. Arrows indicate L: liver and B: bladder. (TIFF) [file pone.0087002.s003.tiff]

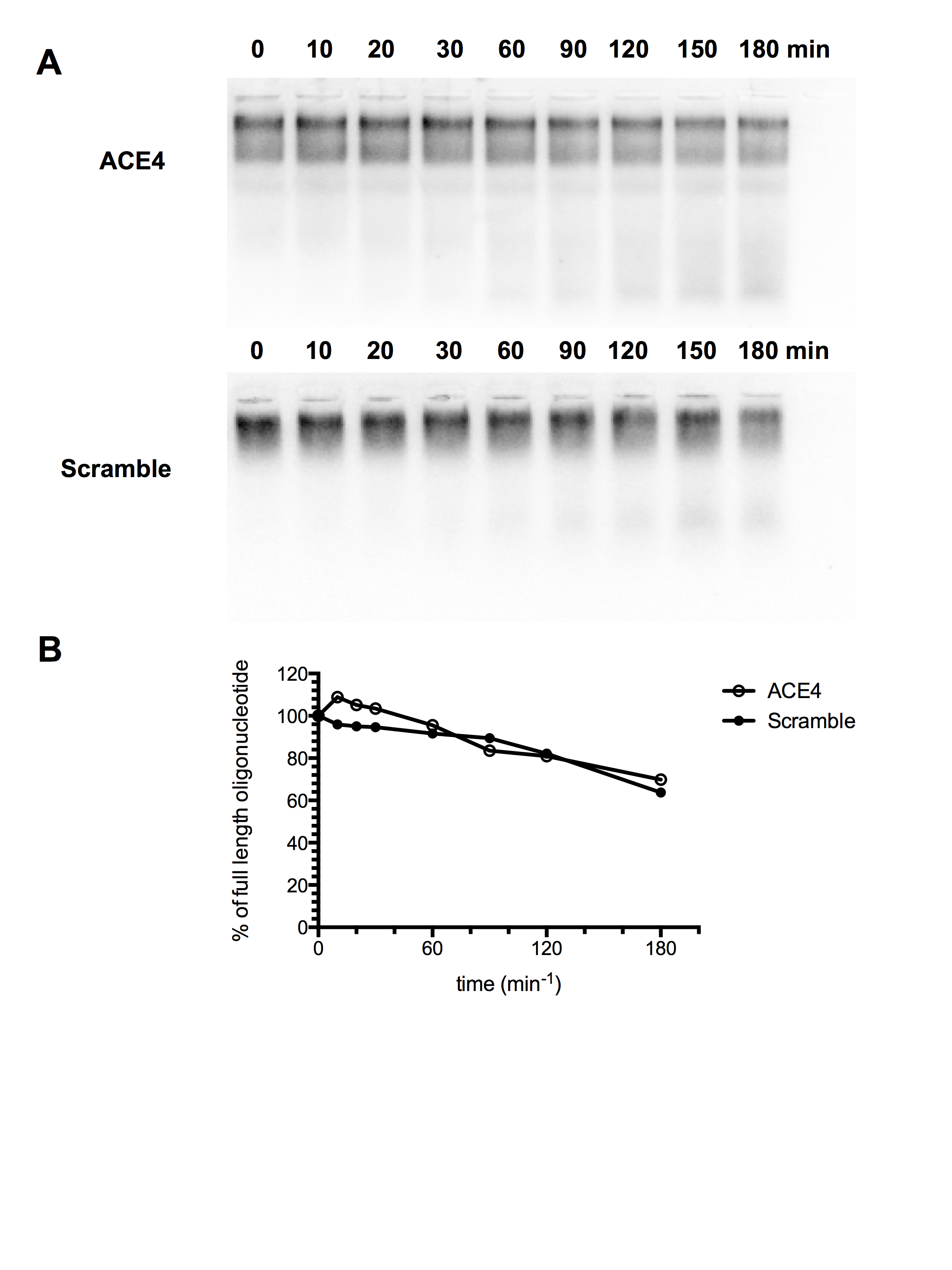

Supplement: Figure S4 — Nuclease resistance of the ACE4 aptamer and the scramble sequence in 10% serum. A) The fluorescent ACE4 aptamer (upper panel) and scramble sequence (lower panel) were incubated at 37°C for different times in 10% serum before being analyzed by electrophoresis on a 3% agarose gel. B) Evolution of the percentage of full-length oligonucleotides over time measured from A). (TIFF) [file pone.0087002.s004.tiff]

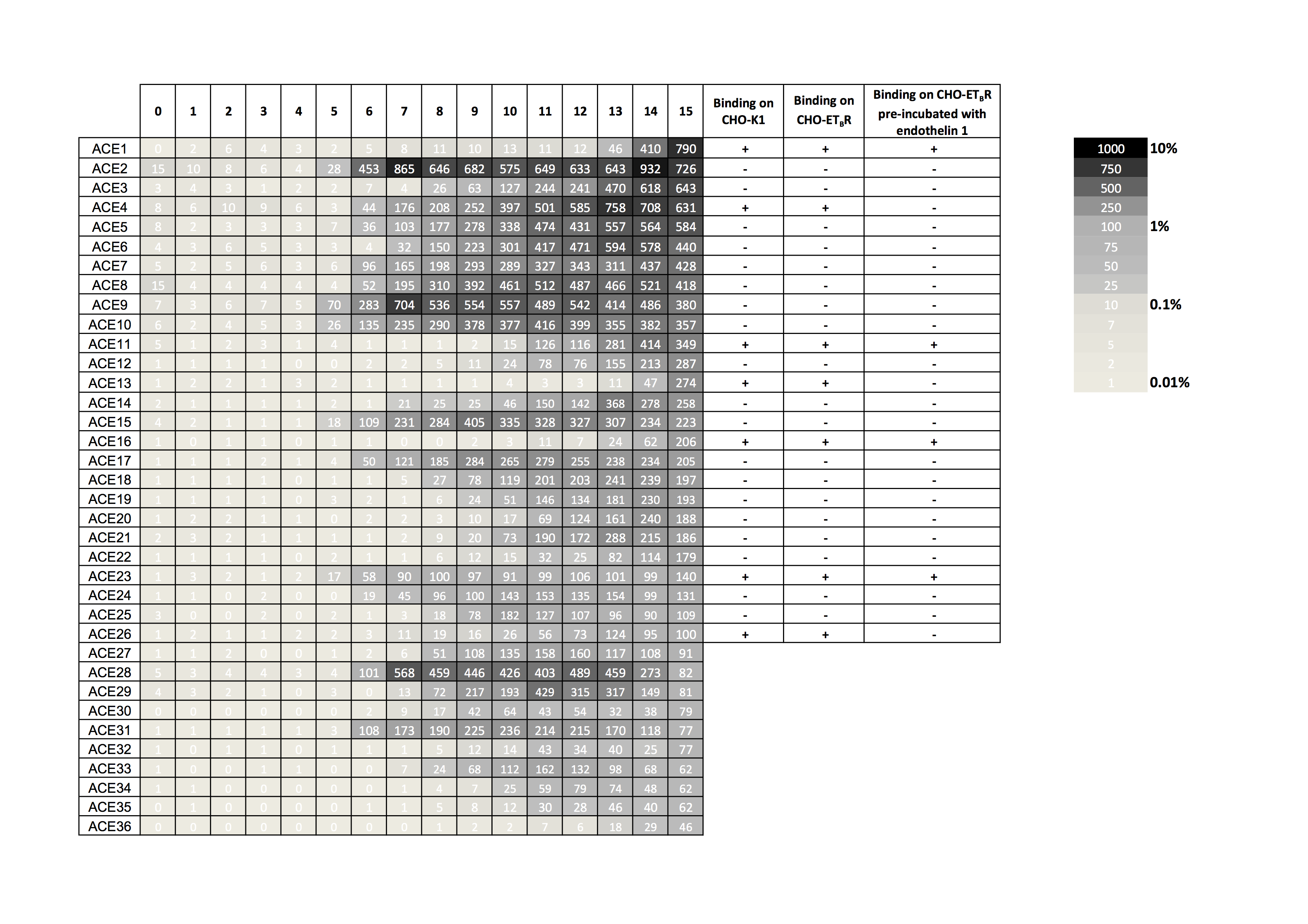

Supplement: Table S1 — Evolution of sequences during the rounds of cell-SELEX. 135 sequences have been found at more than 0,05% of the pool during at least one round of cell-SELEX. They are ranked from their relative abundance in the latest round and their numbers per 10,000 sequences of the pool are presented for each round. Binding at 25 nM on CHO-K1 cells, CHO-ETBR cells and CHO-ETBR cells pre-incubated with Endothelin-1 was evaluated for the sequences that represent more than 1% of the pool after the last round,+indicates binding, – indicates no significant binding compared to a scramble sequence. (TIFF) [file pone.0087002.s005.tiff]
